# Pig Genomics and Genetics

**DOI:** 10.3390/genes12111692

**Published:** 2021-10-25

**Authors:** Katarzyna Piórkowska, Katarzyna Ropka-Molik

**Affiliations:** National Research Institute of Animal Production, Animal Molecular Biology, 31-047 Cracow, Poland; katarzyna.ropka@iz.edu.pl

The pig (Sus scrofa) is the most popular large farm animal in the world. They are frequently used as animal models for human medical research due to high biological similarity to humans, such as body proportions [1,2], metabolic process [3,4], adipose tissue distribution and adipocyte size [5]. In addition, both species reveal also a high genetic analogy: the human genome is composed of 3.5 billion bp, the pig genome of 3.0 billion bp; 21,630 protein-coding genes were identified in pigs, while in humans, this is 20,310 [6,7].

On the other hand, molecular biology methods assist agricultural progress, for example, in pig production and breeding. In addition, since the reference genome sequence of the domestic pig was assembled in 2012, the identification processes of crucial phenotypic traits and search of genetic markers for selection have been significantly refined, including the newest wide-range high-throughput techniques. The use of these new genomic tools has the advantage of generating information about multiple genes and gene products in parallel, which makes it possible to identify pathways and gene interactions [8,9]. This approach provides insight into the epistatic effects of genes that could improve understanding of the genetic component of pig phenotype. At first, DNA microarray that is broadly used to date, supports livestock production by predicting the potential genetic breeding value of farm animals [10]. Microarray approach also serves as a research tool in pig breeding, as well. For example, Lee et al. [11] used it to prove that the porcine immune system was affected by different breeding environments, suggesting the importance of controlling microbes in the animal room for qualified research. Another kind of microarray is used to identification of gene expression. In the Sun et al. [12] study, the authors applied cDNA microarray to identify differentially expressed genes (DEGs) between two Chinese pig breeds, pinpointing the association between BAX and BMPR1B genes with litter size. Such methodology allows to highlight potential genetic markers which can used in the pig industry. However, this method, due to numerous limitations in data analysis [13] and the possibility to identify only profiles predefined transcripts/genes through hybridization [14], is eagerly replenished by ‘omic’ approaches. Omic methods integrate structural and functional genomics and relate them with phenotypic data for farm animals, including pigs [8]. They offer the comprehensive detection of the whole transcriptome, genome, proteome, etc. [15]. Next-generation sequencing (NGS) methods using high throughput platforms identify genetic and transcriptomic components by sequencing long hundred-nucleotide reads and then mapping them to the reference genome [15]. Using this approach, Piórkowska et al. [16] pinpointed a new gene cluster involved in porcine meat quality determination via regulating cell proliferation and differentiation and calcium-binding. In turn, more advanced tool PacBio sequencing platform providing ultra-long sequencing reads, allow in the more precise manner identifying gene mutations, new transcripts and gene candidates throughout the whole genome, transcriptome, or epigenome and estimating quantitative traits important for breeding as well as the genetic backgrounds of inherited diseases. However, PacBio is a very expensive method and for now it is applied mainly to improve genome reference, also included pig genome [17].

In this Special Issue, we will present the state of the art in the field of pig genetics and genomics, including the identification of gene candidates linked to important pig traits and to nutritional modifications, with the aim of collecting the most recent advances. Manuscripts focusing on high-throughput methodologies, such as RNA sequencing, ATAC-seq, MACE-seq, chip-seq and RRBS and covering other fields of pig genetics are included.
